# Patient-Reported Outcome (PRO) Consortium translation process: consensus development of updated best practices

**DOI:** 10.1186/s41687-018-0037-6

**Published:** 2018-02-27

**Authors:** Sonya Eremenco, Sheryl Pease, Sarah Mann, Pamela Berry, on behalf of the PRO Consortium’s Process Subcommittee

**Affiliations:** 1 Patient-Reported Outcome (PRO) Consortium, Critical Path Institute, 1730 East River Road, Suite 200, Tucson, AZ 85718-5893 USA; 2 Outcomes and Evidence, Global Health and Value, Pfizer Inc, NYC, USA; 3 Janssen Global Services LLC, Horsham, PA USA

**Keywords:** Patient-reported outcome, Translation, Cultural adaptation, Linguistic validation, Universal translation, Country-specific translation

## Abstract

This paper describes the rationale and goals of the Patient-Reported Outcome (PRO) Consortium’s instrument translation process. The PRO Consortium has developed a number of novel PRO measures which are in the process of qualification by the U.S. Food and Drug Administration (FDA) for use in clinical trials where endpoints based on these measures would support product labeling claims. Given the importance of FDA qualification of these measures, the PRO Consortium’s Process Subcommittee determined that a detailed linguistic validation (LV) process was necessary to ensure that all translations of Consortium-developed PRO measures are performed using a standardized approach with the rigor required to meet regulatory and pharmaceutical industry expectations, as well as having a clearly defined instrument translation process that the translation industry can support. The consensus process involved gathering information about current best practices from 13 translation companies with expertise in LV, consolidating the findings to generate a proposed process, and obtaining iterative feedback from the translation companies and PRO Consortium member firms on the proposed process in two rounds of review in order to update existing principles of good practice in LV and to provide sufficient detail for the translation process to ensure consistency across PRO Consortium measures, sponsors, and translation companies. The consensus development resulted in a 12-step process that outlines universal and country-specific new translation approaches, as well as country-specific adaptations of existing translations. The PRO Consortium translation process will play an important role in maintaining the validity of the data generated through these measures by ensuring that they are translated by qualified linguists following a standardized and rigorous process that reflects best practice.

## Background

The PRO Consortium was formed in 2008 by the Critical Path Institute (C-Path) in cooperation with the U.S. Food and Drug Administration’s (FDA) Center for Drug Evaluation and Research and the pharmaceutical industry [1, 2]. Its mission is to establish and maintain a collaborative framework with appropriate stakeholders for the qualification [3] of patient-reported outcome (PRO) measures and other clinical outcome assessment (COA) tools that will be publicly available for use in clinical trials where COA-based endpoints are used to support product labeling claims.

The PRO Consortium’s structure consists of a Coordinating Committee, subcommittees that address consortium-wide topics, and therapeutic area working groups. The Coordinating Committee, including one C-Path representative and representatives from each member firm, oversees consortium operations, recommends research priorities, and approves research projects and policies, among other functions. Among all the subcommittees, the Process Subcommittee is charged with developing policies and procedures that address common concerns and issues that have implications for all PRO Consortium working groups, identifying areas for further research to advance measurement science, and identifying needs for guidance on procedural issues.

Currently, there are 10 working groups in the PRO Consortium focusing on diseases or conditions with an unmet measurement need. The goal of these working groups is to generate and/or compile the necessary evidence to enable new or existing COA measures to be qualified by FDA for use in assessing primary or secondary clinical trial endpoints.

The PRO Consortium has developed a number of novel PRO measures through its therapeutic area working groups. Once qualified by FDA, these measures could, over time, become ‘gold standard’ assessment tools for a given concept of interest and context of use. It is therefore imperative that the integrity of these measures be maintained when they are implemented in clinical trials and other research studies. Clinical trial sponsors (e.g., pharmaceutical firms) seeking to use these newly developed PRO measures frequently require translation and cultural adaptation (TCA) of the tools for use in global clinical development programs, which together with cognitive interviewing of the resulting translations is known as linguistic validation (LV) [4]. The PRO Consortium defines LV as the process of assessing and confirming the conceptual equivalence [4, 5] and content validity of translations of PRO measures. The Process Subcommittee therefore determined that a detailed LV process was required to ensure that all translations of Consortium-developed PRO measures are performed using a standardized approach with the rigor required to meet regulatory and pharmaceutical industry expectations, as well as having a clearly defined instrument translation process that the translation industry can support.

Numerous translation companies perform LV. However, during the development of the International Society for Pharmacoeconomics and Outcomes Research (ISPOR) principles of good practice for the TCA of PRO measures [6], members of the ISPOR TCA Task Force found that translation companies often employed differing methods for similar translation/adaptation tasks and used different terminology when referring to the same aspects of the translation process, all of which could increase variability between languages and potentially undermine the validity of research data collected with these measures, as well as the aggregation of global data sets. Based on their findings, a high-level set of consensus-based good practices was developed [6], hereafter referred to as ISPOR recommendations. Published in 2005, the ISPOR recommendations present a 10-step translation and cognitive interview process; this document is frequently referenced as a recognized LV methodology within the pharmaceutical industry and by FDA. Since its publication, literature reviews have been published which compare LV approaches found in published guidelines [5, 7]. Epstein and colleagues [7] found that there was no consensus in the literature reviewed and recommended that any validated method could be used, while Acquadro and colleagues [5] found that most guidelines recommend a multistep process, but varied regarding the actual steps necessary. In both cases, the authors recommended that empirical research on LV methodology is needed rather than expert-based guidelines.

We acknowledge that no ‘gold standard’ for LV of PRO measures exists [7] due to a lack of empirical evidence to support one approach over another. The Process Subcommittee decided to use the ISPOR recommendations as a starting place for the consensus process rather than to conduct additional literature reviews given the significant variability in methods already noted. The Process Subcommittee also sought an efficient method for finalizing the PRO Consortium LV process to facilitate the completion of required translations, which did not allow for the conduct of a comparative study.

While the ISPOR recommendations [6] remain relevant, the Process Subcommittee determined that the recommendations do not provide the level of detail required by end users to ensure consistency in the LV process when different translation companies are commissioned to translate PRO measures. Additionally, it is important to ensure that best practice guidelines are updated periodically to reflect any methodological advances and changes in regulatory requirements since their development. The ISPOR recommendations had been in use for a decade when the PRO Consortium’s consensus process began, and they were based on literature and experience dating back 10 years or more prior to publication. Recent feedback from representatives from clinical trial sponsors and translation companies confirms that current LV methodologies continue to differ across companies. The ISPOR recommendations were intentionally not prescriptive with regard to how the steps were performed, and as a result, member firms interested in using PRO Consortium measures in their trials requested further guidance regarding the appropriate LV process for these measures. As such, this consensus process was also driven by the need to meet increasing clinical trial requests. With the goal of ensuring consistency in the implementation of PRO Consortium-developed measures in global clinical trials and in the methods and evidence used to support their LV, the Process Subcommittee determined it would be necessary to develop a detailed LV process that reflects current best practice based on the ISPOR recommendations with adequate detail to ensure that a standardized LV methodology is used for all PRO Consortium measures.

The Process Subcommittee’s initial goal was to define a detailed LV process that reflects the rigor that regulatory agencies expect without being excessively burdensome for translation companies to perform. The FDA’s guidance for industry titled Patient-Reported Outcome Measures: Use in Medical Product Development to Support Labeling Claims (hereafter called FDA’s PRO Guidance) [8] refers to the importance of comparability of content validity and other measurement properties across all language versions of a PRO measure. Although FDA’s PRO guidance does not specify LV requirements for individual situations, it states that FDA will review methodologies and documentation surrounding steps that were taken to prepare translations for populations that will be included in clinical trials. In addition, the European Medicines Agency (EMA) in its reflection paper on the regulatory guidance for the use of health-related quality of life (HRQL) measures in the evaluation of medicinal products [9] states that it will evaluate the strength of evidence of validation including cultural adaptation/translation as applicable to the study. Awareness of these regulatory recommendations remained at the forefront of the PRO Consortium’s LV process development. Given the time that has passed since the release of the ISPOR recommendations [6] and EMA reflection paper [9] in 2005 and the FDA’s PRO Guidance in 2009 [8], the Process Subcommittee felt it would be beneficial to review and evaluate the ISPOR recommendations, possibly supplement them, and provide updates based on feedback solicited from translation companies and pharmaceutical industry experts. The ultimate goal was to create a translation process where content validity and other measurement properties are comparable to the original language version.

There is not always a clear path forward when determining translation needs based on regulatory guidance. Based on feedback from translation agencies and clinical trial sponsors, the Process Subcommittee considered it would be helpful to the pharmaceutical industry, the translation industry, and regulatory agencies to provide a uniform translation process as a reference. This would allow alignment on the topic and a basic understanding of good LV practices.

This paper is meant to complement the ISPOR recommendations and focuses on improvement in qualitative methods for translation and LV by providing additional detail, steps, and information to clinical trial sponsors and to translation companies. This paper summarizes the consensus process used to review and expand upon the ISPOR recommendations and outlines the resulting steps in the PRO Consortium-defined LV process. The final translation process documents are currently in use as guideline documents for member firms that are funding new language translations of PRO Consortium measures for use in their multinational trials.

### Consensus process

## Methods

The PRO Consortium followed a structured, iterative feedback process to reach consensus on its LV methods. The process was similar to a modified Delphi approach [10, 11] in that information was gathered from individuals representing companies experienced in LV as well as researchers with this expertise, and their information was consolidated and returned to the group members for further comment and feedback in two iterative rounds. The process differed in that each individual’s feedback from each round was not shared with group members in successive rounds, but rather adjudicated by the PRO Consortium team (consisting of the Executive Director, two Project Managers, an LV expert, and an external measure development consultant) to prepare revised documents for the following round, and there was no final vote taken to reach consensus. Group members were asked whether they had strong objections to accepting the proposed process in the final feedback round. The LV process was finalized at the point at which no further objections from either the translation company representatives or the pharmaceutical industry representatives were raised.

In 2015, the PRO Consortium initiated the development of an instrument translation process by requesting information from 13 translation companies, members of the Process Subcommittee, and other LV experts to determine whether the ISPOR recommendations were being followed and to identify additional best practices within the translation industry that exceeded the ISPOR recommendations. This information-gathering process involved reviewing documentation (e.g., guidelines, websites) from the 13 translation companies and conducting follow-up discussions via telephone or email to clarify the exact LV methods used by each company. A matrix document was created which listed the steps from the ISPOR recommendations, and then each company’s version of the steps to facilitate comparison of the execution of each step, which resulted in a total of 17 steps. Individual translation company procedures were compared with each other and against the ISPOR recommendations to identify the most rigorous approach for each suggested step in the process based on PRO Consortium team determination. A document was created that listed 17 steps in three columns: the ISPOR steps in column 1, the most rigorous steps identified in our review in column 2, and then the proposed PRO Consortium version of each step in column 3. Rather than looking for the most commonly performed way of executing each step, this approach was intended to identify the best practice approach for each step. The document was then reviewed by an LV expert to ensure that regulatory needs would be met by the proposed PRO Consortium steps and to identify steps that were either company-specific and not required as part of best practice or not necessary for regulatory purposes. This review led to the removal of four steps which were either deemed too burdensome and not required as part of best practice (i.e., risk mitigation assessment and plan, risk audit and summary report) or not the responsibility of the translation company (i.e., establishing a document repository, translatability assessment), and consolidation of two steps into one due to redundancy (forward translation reconciliation and establish reconciled forward translation).

In early 2016, the PRO Consortium used the 12 steps identified during the LV best practices information-gathering stage to draft a proposed Instrument Translation Process in the form of a numbered table that described each recommended step in moderate detail. The proposed process was distributed to the original 13 translation companies along with three others identified in 2016 which perform LV of PRO measures and were known to PRO Consortium member firms. In an effort to reach consensus, the translation companies were asked to review the proposed process document and provide constructive feedback including, if applicable, an indication of agreement with the process described. Recommendations on ways to improve the delineation of the process or identification of areas of concern were also requested. This round of review took place between March and May 2016, with written responses received from 13 of the 16 (81%) companies invited to participate. Concerns were raised regarding most steps and/or descriptions in the process. These concerns were reviewed by the LV expert and then adjudicated by the Process Subcommittee.

Based on feedback received from translation companies during the initial round of review, a number of significant changes were made to the process, additional process documents were prepared in response to needs identified in the feedback round, and these documents were then circulated for review and comment by translation company representatives and Process Subcommittee representatives in July 2016.

A consensus development teleconference was held in August 2016, attended by nine translation company representatives and four Process Subcommittee members, during which further feedback on the revised process documents was discussed by attendees. Additional revisions were made following the teleconference to address the remaining issues and final versions of the process documents were reviewed by the Process Subcommittee and formally approved by the PRO Consortium’s Coordinating Committee. The documents are now available for use by PRO Consortium member firms as well as the broader scientific community.

### Changes to the PRO consortium instrument translation process based on input from multiple stakeholders

**Fig. 1:**
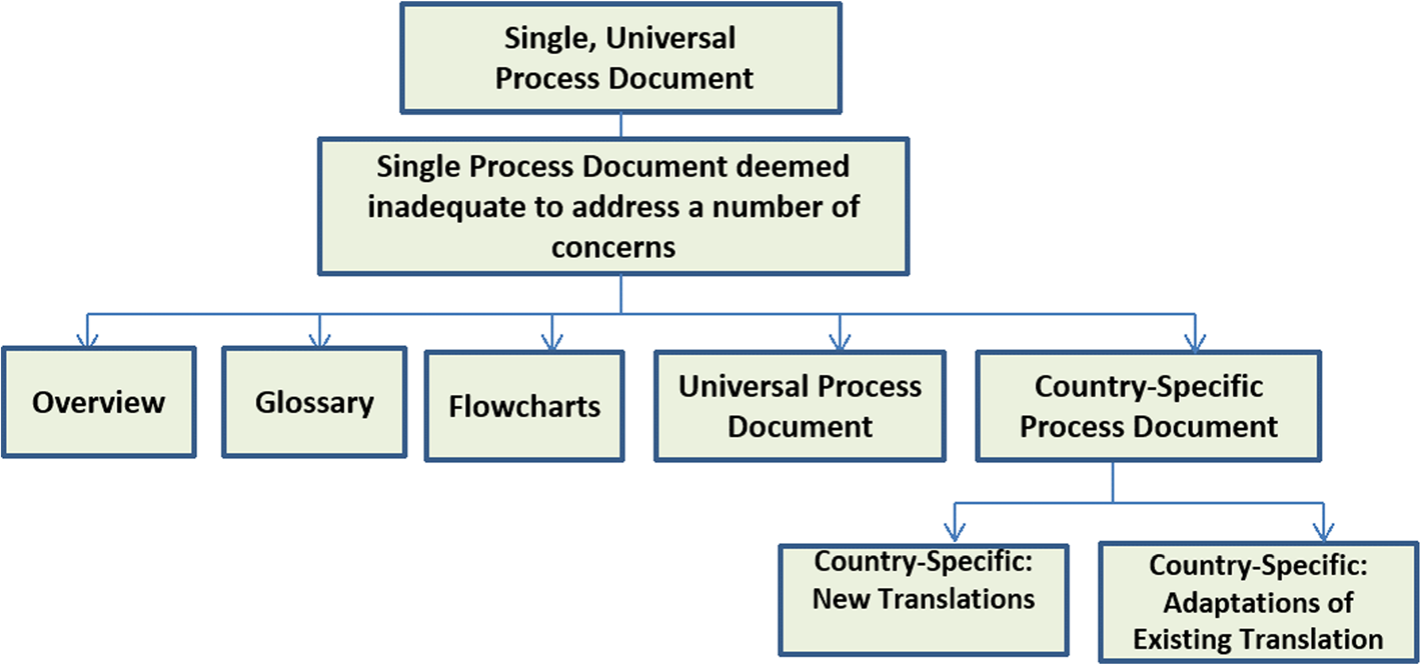
Overview of PRO Consortium Translation Process Development

A number of changes were made to the PRO Consortium’s instrument translation process based on the feedback received from translation companies and member firms. As shown in Fig. 1, the single process document originally distributed was inadequate to delineate the process envisioned by the PRO Consortium, and led to the creation of several separate documents to explain the PRO Consortium Instrument Translation Process: an overview, glossary, flowcharts, and separate process documents to address universal and country-specific approaches (See Table 1. Definitions of Key Terms for definitions). The country-specific process was separated into two documents, one for new translations and another for adaptations of an existing translation.

**Table 1:** Definitions of Key Terms

Key Term	Definition
Back-translation	Process of translating a document that has already been translated into another language back to the original language - preferably by an independent translator.
Country-specific translation	Translation approach focused on respecting the diversity of languages and sense of cultural identity of the target populations through their languages. Involves preparing separate translations for countries sharing the same language (e.g., separate Spanish translations for Spain, Mexico, Chile, and U.S.).
Forward translation	Translation from the source language to the target language.
International Harmonization	Harmonization of all translations with each other and the source version. Objective is to detect and deal with any discrepancies between different language versions that threaten conceptual equivalence and comparability across languages. Provides an additional quality control step and further ensures that data from global trials can be safely aggregated.
Linguistic validation	The process of assessing and confirming the conceptual equivalence [4, 5] and content validity of translations of patient-reported outcome (PRO) measures. Usually, linguistic validation refers to a process whereby translated text is actively tested with patients in the target population and target language group through cognitive interviews.
Preparation	Initial planning and actions carried out before the translation process begins, such as identifying translation consultants and in-country affiliates and creating translation files if needed.
Forward translation reconciliation	Process of comparing and merging more than one forward translation into a single forward translation resulting in a reconciled forward translation.
Universal translation	Translation approach focused on commonalities rather than differences to develop one version to be used in regions or countries speaking the same language (e.g., preparing a single Spanish translation that will be used by all Spanish-speaking countries).

Other significant changes to the PRO Consortium Translation Process included the following:

#### Universal vs country-specific approaches

Although most translation companies perform both types of translations, six supported the PRO Consortium’s preference for universal translations, while seven raised questions or expressed concerns with the approach. Change: The ‘Preparation Step’ was revised to state that C-Path, in conjunction with the clinical trial sponsor, will determine if the translations to be performed will follow the universal or country-specific approach.

#### Use of certified translators

Five translation companies disagreed with this requirement, noting that many of the best translators are not certified and that certification is not available in some countries. Change: Reference to ‘certified’ was replaced with ‘qualified’ to emphasize the need for translators with appropriate qualifications to perform the translations as opposed to having people without proper training translate PRO measures.

#### Forward translations

Forward translations are handled differently by many translation companies and a minimum number had not been defined. Change: The ‘Forward Translation Step’ was revised to reflect a minimum requirement of two forward translations.

#### Forward translation reconciliation

The reconciliation process is handled differently by translation companies and the approach initially proposed (independent third party reconciles the two forward translations) was considered too complex by many of them. This finding is consistent with Koller and colleagues [12], who provide recommendations for decision-making during reconciliation but did not find consensus on the people involved in the process. Change: In the ‘Forward Translation Reconciliation Step,’ multiple options are provided as examples for accomplishing this process, noting that the decision-making must be well-documented.

#### In-country consultants

Several translation companies expressed concerns and some confusion regarding the involvement of in-country consultants while others strongly supported their involvement. Change: Clarified roles on process documents and in Glossary.

#### Harmonization

There was general confusion regarding what this involved. Change: This step was relabeled the ‘International Harmonization Step’ and requested only a description of the planned methodology and rationale.

#### In-country affiliates

Several translation companies questioned whether in-country affiliates should be involved in conjunction with the proofreading step prior to cognitive interviewing, while others felt that their involvement early in the process was very important. Change: This step was made mandatory to provide affiliate representatives with a recognized role in the process and to prevent unsolicited feedback after translations were finalized that could threaten their validity, and a recommendation was added for the clinical trial sponsor to identify affiliates early (during ‘Preparation’), and that, if none are available, alternatives would be provided by the translation company.

#### ePRO translations

Many translation companies questioned how electronic PRO (ePRO) implementation should be integrated into the process. Change: Addressed in ‘Final Review and Documentation (Proofreading) Step’ as recommended best practice.

#### Clinician review

This was suggested by one translation company and one member firm as a new step. Decision: The sponsor can include this step if desired but it is not required in the PRO Consortium translation process.

Detailed descriptions of the Steps mentioned above are available in Table 3.

### Documents generated

Table 2 presents the current set of process documents that describe the PRO Consortium translation process. The Overview was developed to provide both clinical trial sponsors and translation companies with the appropriate context in which to perform LV and awareness of the overall purpose and objectives. We also thought it was necessary to review and clarify terms that are currently being used for LV and created an updated Glossary to be used for consistency across the translation industry.

Given the feedback from translation companies and PRO Consortium member firms, it was decided that the PRO Consortium process needed to allow flexibility regarding whether a given measure or language would follow the universal or country-specific approach [4, 13] and, as a result, detailed descriptions of each process were developed. The Process Subcommittee along with member firms and translation companies worked together to discuss the pros and cons of universal and country-specific translations. The PRO Consortium generally prefers universal language translations for its measures to reduce logistical complexity of managing country-specific versions and minimize variability among translations of the same language but also recognizes that there are some situations where the universal approach is not optimal. For example, health resource utilization varies by country, so translations of these questionnaires must be tailored to the country in question and cannot be translated universally. Flexibility is recommended to accommodate situations where wording of the measure (requiring different terms in different countries) or the language in question (which may vary too much between countries) does not allow universal translations to be utilized. Flowcharts for each of the three processes were developed to provide a visual representation of the sequence of the steps.

Finally, the process steps emphasize the importance of documenting the rationale for decision-making throughout the LV process, which would be compiled in a report on the LV process and results by the translation company. The PRO Consortium created a report template to facilitate standardized reporting across translation companies.

**Table 2:** PRO Consortium Instrument Translation Process Documents

Process Document	Description
Translation Process Overview	Provides information regarding the PRO Consortium, measure development, use of measure, instrument translation process goals, and repository that will be maintained of translation documents.
Glossary	Defines terms, roles, and documents included in the process documents.
Step-by-step translation process documents: 1. Universal 2. Country-specific – New language 3. Country-specific – Adaptation of existing language	Provides detailed descriptions of steps/sub-steps to be followed for each of the three types of translations.
Translation process flowcharts (3)	Identifies steps to be followed for each of the three types of translations in a visual flow.
PRO Consortium Instrument Translation Report template	For reports prepared by translation companies for PRO Consortium measures.

*PRO*: patient-reported outcome

### Translation process steps

This section outlines the steps in the PRO Consortium translation process and describes aspects that differentiate this process from the ISPOR recommendations. The process outlined here is intended for use with PRO measures, which make up the majority of measures in development by the PRO Consortium. In cases where a different COA (e.g., clinician-reported outcome, observer-reported outcome, or performance outcome) measure might be developed, the translation process will be reviewed and refined to address the new assessment type.

Table 3 presents the 12 steps in the translation process and provides a brief description of how each is implemented for both the universal and the country-specific approaches. The country-specific adaptation process can occur either within the same study if relevant countries are included initially or subsequent to completion of the initial translation, if an existing translation needs to be adapted for a new country identified later.

**Table 3:** PRO Consortium Translation Process Steps

Step Number	Step Name	Universal Approach	Country-specific Approach for New Language
1	Preparation	Obtain permission to translate, decide on approach, and Item Definition Table provided. Translation consultants identified for each of the target countries. In-country affiliates identified or back-up option if necessary. Plan for final review and proofreading in the mode to be used in the clinical trial and whether additional text (e.g., error messages, navigational terms) needs translation in addition to the measure itself.	Obtain permission to translate, decide on approach, and Item Definition Table provided. “Mother”^a^ country selected and translation consultants identified for “Mother” country and for adaptations if required. In-country affiliates identified or back-up option if necessary. Plan for final review and proofreading in the mode to be used in the clinical trial and whether additional text (e.g., error messages, navigational terms) needs translation in addition to the measure itself.
2	Forward Translation	Minimum of 2 forward translations by translators from different target countries if applicable.	Minimum of 2 forward translations by translators from “Mother” language.
3	Reconciliation	Forward translations are reconciled into one translation, with several options to accommodate translation company practices. Universal approach seeks to find a solution that works across target countries. Rationale documented.	Forward translations are reconciled into one translation in “Mother” language, with several options to accommodate translation company practices. Rationale documented.
4	Back-translation	Conduct at least one back-translation of the reconciled forward translation. (Back-translator to be kept blind to source questionnaire and Item Definition Table.)	Conduct at least one back-translation of the reconciled “Mother” forward translation. (Back-translator to be kept blind to source questionnaire and Item Definition Table.)
5	Revision of Reconciled Forward Translation	Evaluate back-translation to assess semantic equivalence [14] and identify issues in the reconciled translation, agree on revisions needed, taking into consideration feedback from multiple target countries, and implement changes.	Evaluate back-translation to assess semantic equivalence [14] and identify issues in the reconciled translation, agree on revisions needed, and implement changes.
5A	Adaptation of “mother” target language for other countries (country-specific only)	Not applicable.	Two parallel reviewers from each target country review “Mother” language version and propose changes to suit their country. Reconciliation of the two adaptations as in Step 3, back-translation of adapted items and evaluation of issues, and revision as needed based on back-translation evaluation.
6	International Harmonization	All languages in the project are reviewed for consistency and conceptual equivalence with each other and the original language version.	All languages in the project are reviewed for consistency and conceptual equivalence with each other and the original language version.
7	Proofreading	Two or more proofreaders from different target countries check translation, and correct any remaining spelling, diacritical, grammatical or other errors; clinician review is optional. In-country affiliate(s) review translation separately.	Two or more proofreaders for “Mother” language and adaptations check translation, and correct any remaining spelling, diacritical, grammatical or other errors; clinician review is optional. In-country affiliate(s) review translation separately.
8	Cognitive Interviewing	Pilot testing and cognitive interviewing conducted in each target country, with a minimum of 5 participants per language/country who match the target population for as many criteria as reasonably practical. In-person where possible. Testing to be done for target language in each target country in the study associated with that language (e.g., for German, conduct pilot testing in both Germany and Austria). If another relevant country is added in the future, additional cognitive interviews with the universal version in the new country (e.g., Switzerland) need to be conducted.	Pilot testing and cognitive interviewing conducted in each target country, with a minimum of 5 participants per language/country who match the target population for as many criteria as reasonably practical. In-person where possible.
9	Post-Cognitive Interview Review (Analysis/Revisions)	Review cognitive interview results and compile feedback for translation team resolution. Agree on any revisions to reconciled forward translation identified during cognitive interviews.	Review cognitive interview results and compile feedback for translation team resolution. Agree on any revisions to reconciled forward translation or adaptations identified during cognitive interviews.
10	Final Review and Documentation (Proofreading)	Ensure proposed revision maintains conceptual equivalence and does not threaten international harmonization for future data pooling purposes, implement revisions, proofread revised translations, and document any relevant alternatives in the Item Definition Table. Conduct final proofreading of measure translations (format/layout) for mode(s) of implementation (e.g., screen shots, paper) to identify any mistakes or errors that may impact integrity of data collection.	Ensure proposed revision maintains conceptual equivalence and does not threaten international harmonization for future data pooling purposes, implement revisions, proofread revised translations or adaptations, and document any relevant alternatives in the Item Definition Table. Conduct final proofreading of measure translations (format/layout) for mode(s) of implementation (e.g., screen shots, paper) to identify any mistakes or errors that may impact integrity of data collection.
11	Report	Prepare final summary report documenting development of each translation and providing description of all translation and cultural adaptation decisions.	Prepare final summary report documenting development of each translation or adaptation and providing description of all translation and cultural adaptation decisions.
12	Archiving/Record-keeping	Documentation to be archived: • Qualifications and experience of translation team • Documentation of changes made throughout the translation work and rationale for changes • Translation certificates • Translation report including results of cognitive interviews	Same as Universal approach.

^a^In a country-specific translation situation where many countries using that language may need to be included in a study, one of the languages referred to as the “Mother” language is chosen to undergo the initial steps of forward translation, reconciliation, and back-translation. At that point, the “Mother” language version can be adapted for use in other countries without having to start from the beginning. An example would be German, where Germany would be chosen as the “Mother” language, and used as the basis for German adaptations for Austria and Switzerland

It is important to note that semantic and conceptual equivalence [14] are goals of the PRO Consortium translation process. The evaluation of conceptual equivalence begins during the measure development process with the mandatory translatability assessment conducted to ensure that the concept of interest and the wording of the items are suitable in cultures outside of North America where the initial measure development was conducted. An Item Definition Table describing the concepts being measured along with translation alternatives is also developed as part of the measure development process, as recommended by Herdman and colleagues [14] for achieving semantic equivalence. Both processes are considered outside the scope of the translation process because they occur during measure development but provide the necessary foundation to improve the semantic and conceptual equivalence of the translations. Both types of equivalence are further evaluated during the translation process as described in Table 3 (see Steps 5 and 6).

The PRO Consortium translation process expands upon the ISPOR recommendations [6] in several ways. One major difference is that the PRO Consortium process takes into account both the universal and country-specific approach and delineates each approach including two variations of the country-specific approach in a very detailed, step-by-step fashion. A later ISPOR Task Force report [13] explored the issue of same language in different countries and discussed the advantages and disadvantages of universal and country-specific approaches at a high level, but it did not provide sufficient detail to be operationalized by the PRO Consortium.

A second way that the PRO Consortium process differs from the ISPOR recommendations is the incorporation of a required affiliate review. Member firms strongly endorsed this step as an important way for their local affiliates to provide input into the translations during the process rather than after the translation has been finalized when changes could contradict the evidence generated to support the translation throughout the LV process. In response to feedback from translation companies that this step could cause delays or result in unnecessary changes, it was refined to include an explanation with specific directions about the nature of the review that would be provided to affiliates so that only critical issues would be raised. In addition, for those firms without affiliates willing to participate in the process, a provision for the translation company to provide a suitable alternative to fill this role was included. For this reason, the process mentions identification of affiliate reviewers during the preparation step very early on to ensure that a determination can be made if alternatives need to be engaged and that all reviewers are aware of the timelines and can respond in a timely way to the request for review.

A third differentiator in the PRO Consortium process is the inclusion of considerations for electronic implementation of the measures and the awareness of its potential impact on the translation process. First, there is a potential need for supplementary text to be translated to accompany the measure when implemented electronically, including skip alerts or other error messages that are not necessary on paper. It is best to identify such text early in the process so that a decision can be made whether to include it in the above-described process or to have it translated in a separate effort along with other navigational text, which does not require the rigorous translation and cognitive interviewing process described here. Second, a critical part of the electronic implementation of the measures is the proofreading of final screenshots to ensure accurate transfer of the translated content to the electronic platform. Ideally, this screenshot proofreading process would be conducted by the same translation company responsible for the translation of the content to prevent unauthorized changes, and therefore this work needs to be included in the translation company’s scope of work. It is also critical for both the translation company and the electronic COA provider to coordinate the screenshot proofreading process to meet study start-up timelines.

Another unique aspect of the PRO Consortium translation process is the decision to encourage use of the same translation company selected for initial measure translations to produce all subsequent translations. The rationale for this recommendation is that using the same company for all translations of a specific measure will ensure that a consistent and appropriate methodology is used across all languages, and that the firm would maintain institutional memory regarding the measure’s nuances and previous decisions leading to increased and improved harmonization of translations developed over time. The goal is to enhance translation company commitment to the quality and accuracy of translations of the measure because of its ongoing involvement in the LV process. This approach will also likely expedite the process for subsequent translations.

There are some limitations to the methods used to develop consensus for the PRO Consortium translation process. The stakeholders involved in the process included the constituents of the PRO Consortium but did not include input from researchers who conduct studies in multiple languages outside of a pharmaceutical sponsor context. Input from such researchers could have provided a useful viewpoint on the proposed process and considerations for improvement. Another limitation is that the PRO Consortium did not conduct any comparison studies to test different methods to provide empirical support for the decisions made about best practices. Studies comparing different translation methodologies have been conducted [15–17] and found that the different translations had similar measurement properties and resulted in reliable measures. To date, better methods to conduct LV comparison or feasibility studies still need to be developed. The PRO Consortium recommends that sponsors review the results of translations used in their clinical trials by language to further evaluate the comparability of measurement properties as an important next step in the evaluation of the translation process. As the translation of several PRO Consortium measures is currently underway, further insights into the feasibility of the process will be evaluated and the process revised if needed.

## Discussion/conclusion

Despite the fact that recommendations for a rigorous approach to translation of PRO measures have been in place for decades [5, 7, 18–22], translations for clinical trials have not followed a consistent methodology and were sometimes performed by local language speakers who were not qualified translators. However, the member firm representatives within the PRO Consortium recognize that this approach can put valuable PRO data at risk of bias or increased variability that can attenuate an efficacy signal. Therefore, the PRO Consortium member firms support, and will benefit from, having a well-defined and clear process for LV of the measures emerging from the Consortium’s working groups, which are intended to serve as primary or key secondary endpoint measures in support of product label claims. The PRO Consortium translation process will play an important role in maintaining the quality of the data generated through these measures by ensuring that they are translated by qualified linguists following an established, consistent, and rigorous process that reflects best practice. The PRO Consortium’s translation process meets the minimum standards recommended by a task force from the International Society of Quality of Life Research (ISOQOL) in that it documents the methods used and includes qualitative evidence (i.e., cognitive interviews) to evaluate the translation [23]. Regulatory agencies are showing increased concern regarding the cross-cultural suitability of PRO measures that generate data used to support clinical trial endpoints [3, 8, 9]. The FDA’s PRO guidance [8] includes an expectation that translation of such measures follows a recognized process and that the measurement properties are comparable across languages, and the EMA’s reflection paper [9] states that evidence supporting cultural adaptation/translation is expected. The PRO Consortium’s translation process is intended to maintain the integrity of its FDA-qualified measures by ensuring that these regulatory standards will be met. It provides a standardized, consensus-driven approach that enhances the rigor of LV for PRO measures and advances measurement science in a multinational clinical trial environment.

## Abbreviations

Abbreviation: Definition
COA: Clinical outcome assessment
C-Path: Critical Path Institute
ePRO: Electronic patient-reported outcome
FDA: U.S. Food and Drug Administration
ISOQOL: International Society of Quality of Life Research
ISPOR: International Society for Pharmacoeconomics and Outcomes Research
LV: Linguistic validation
PRO: Patient-reported outcome
TCA: Translation and cultural adaptation
U.S.: United States
